# Equivalency between the shock index and subtracting the systolic blood pressure from the heart rate: an observational cohort study

**DOI:** 10.1186/s12873-020-00383-2

**Published:** 2020-10-31

**Authors:** Yohei Kamikawa, Hiroyuki Hayashi

**Affiliations:** 1 Department of Emergency Medicine, University of Fukui Hospital, 23-3 Matsuoka Shimoaizuki, Eiheiji-cho, Yoshida-gun, Fukui, 910-1193 Japan; 2 Department of General Medicine, University of Fukui Hospital, 23-3 Matsuoka Shimoaizuki, Eiheiji-cho, Yoshida-gun, Fukui, 910-1193 Japan

**Keywords:** Ambulance, Blood pressure, Cohort study, Critical illness, Heart rate, Shock index, Tertiary care hospital, Vital signs

## Abstract

**Background:**

Although the shock index is known to predict mortality and other severe outcomes, deriving it requires complex calculations. Subtracting the systolic blood pressure from the heart rate may produce a simple shock index that would be a clinically useful substitute for the shock index. In this study, we investigated whether the simple shock index was equivalent to the shock index.

**Methods:**

This observational cohort study was conducted at 2 tertiary care hospitals. Patients who were transported by ambulance were recruited for this study and were excluded if they were aged < 15 years, had experienced prehospital cardiopulmonary arrest, or had undergone inter-hospital transfer. Pearson’s product-moment correlation coefficient and regression equation were calculated, and two one-sided tests were performed to examine their equivalency.

**Results:**

Among 5429 eligible patients, the correlation coefficient between the shock index and simple shock index was extremely high (0.917, 95% confidence interval 0.912 to 0.921, *P* < .001). The regression equation was estimated as sSI = 258.55 log SI. The two one-sided tests revealed a very strong equivalency between the shock index and the index estimated by the above equation using the simple shock index (mean difference was 0.004, 90% confidence interval 0.003 to 0.005).

**Conclusion:**

The simple shock index strongly correlated with the shock index.

## Background

The shock index (SI) is an indicator of the severity of hypovolemic shock and is calculated by dividing the heart rate (HR) by systolic blood pressure (SBP) [1]. It serves to predict the mortality, need for blood transfusion, or necessity of intensive care unit admission among patients with trauma [2–7], postpartum haemorrhage [8, 9], acute myocardial infarction [10, 11], stroke [12, 13], sepsis [14, 15], and other critical conditions [16, 17]. Numerous previous studies have demonstrated that the SI demonstrates superior prediction for mortality to traditional vital signs, although it has some limitations, including its low sensitivity especially for the elderly or obstetric patients [2–17]. However, in clinical practice, calculating the SI for all patients is difficult. An SI value > 0.9 is generally accepted as a cut-off point for an increased risk of mortality [16], but it is sometimes difficult to quickly calculate whether the patient meets this cut-off when the value of the quotient (particularly when considering the second decimal place) is extremely close to 0.9 (e.g. When a patient has an HR of 103 beats per minute and SBP of 114 mmHg, the quotient is approximately 0.904 and it technically meets the cut-off but it is exceedingly difficult to calculate promptly without a calculator). Having a confusing cut-off value, the SI needs to be interpreted from several variables to identify patients with a critical status but stable HR and SBP making it an impractical indicator that is rarely used in scoring systems assessing emergencies. Furthermore, despite its utility, there is no established consensus on when and where to utilize SI in emergency departments (EDs) [18]. Recent studies have attempted to validate revised SI measurements meant to improve its ability to predict mortality [19–22]; however, such calculations are more complicated and tend to be avoided by clinicians. If the calculation of the SI can be made simpler, it would lead to rapid progress in terms of the clinical research using SI.

Considering that SI is used to represent the different dynamics of HR and SBP [23, 24], it is possible that simply subtracting the SBP from the HR may provide a useful substitute for SI, improving the availability of a calculated value as it is easier to mentally subtract integers than to divide them.

In this study, we identify the proposed new index the simple shock index (sSI) and investigated whether the sSI predicted SI equivalently among patients transported to hospitals via ambulance.

## Methods

### Study design and setting

This observational cohort study was conducted at two urban tertiary hospitals that annually receive via ambulance transport patients (> 2500 and > 4000 respectively). Written informed consent was waived because of the retrospective observational nature of the study, which was conducted using the opt-out method on the hospital websites. All data were fully anonymized. The institutional ethical review board of the University of Fukui Hospital (20160131) and the Fukui Prefectural Hospital (16–60) approved the study’s protocol. All methods were carried out in accordance with relevant guidelines and regulations.

Patients were considered eligible if they were transported to either hospital via ambulance between July 1, 2015 and June 30, 2016. Patients who were aged < 15 years, experienced prehospital cardiopulmonary arrest, or underwent inter-hospital transfer were excluded.

### Study protocol

The collected data included HR in the ED, SBP in the ED, age, sex, trauma, pregnancy status, acute myocardial infarction, sepsis, chronic respiratory disease (previous history of chronic obstructive pulmonary disease, chronic bronchitis, asthma, bronchiectasis, interstitial pneumonia, pulmonary tuberculosis, or lung cancer), and intracranial disease (having suffered from stroke, transient ischemic attack, encephalitis, encephalopathy, seizure, brain tumour, hydrocephalus, concussion, cerebral contusion, or traumatic subarachnoid haemorrhage at arrival to ED). These specific patient characteristics were included since many previous studies have examined the ability of the SI to predict mortality or other critical conditions in those with trauma, pregnancy, acute myocardial infarction, sepsis, and intracranial disease [2–15], and because HR and SBP of aged or chronic respiratory disease patients are known to exhibit specific dynamics [25–27].

HR and SBP were documented immediately following a patient’s arrival to ED. These data were extracted from the electronic medical records. When available, prehospital vital signs documented in emergency service records were used to substitute for missing ED vital sign data. The bedside monitor models BSM-3562 (NIHON KOHDEN, Tokyo, Japan) and PVM-2703 (NIHON KOHDEN, Tokyo, Japan) were used to measure prehospital and in-hospital vital signs, respectively. Any remaining missing data were complemented using the multiple imputation method [28, 29]. To minimize selection or operator bias, all data were collected retrospectively and were fully anonymized before analysis.

The SI and sSI were calculated from the HR and SBP as mentioned above (SI = HR/SBP; sSI = HR − SBP).

### Statistical analysis

Categorical variables were reported as numbers and percentages, while continuous variables were reported as the median and interquartile range (IQR). Patients aged 65 years and older were classified as aged individuals according to the definition widely adopted in developed countries [30]. We used a generally accepted consensus and the 2018 European Society of Cardiology/European Society of Hypertension Guidelines for the management of arterial hypertension [31, 32] to classify patients into four categories according to their SBP as follows: those with SBP of < 90 mmHg were included in the hypotension group, those with SBP of 90–139 mmHg were included in the normotension group, those with SBP of 140–179 mmHg were included in the hypertension group, and those with SBP ≥180 mmHg were included in the severe hypertension group.

First, a correlation plot for SI and sSI derived from all subjects was constructed, and the Pearson’s product-moment correlation coefficient was calculated. Regression analysis was performed using the least-squares method where possible. According to the regression equation, the sSI value, which corresponds to the SI value of 0.9, was determined.

Next, an equivalence test with two one-sided test (TOST) was used to examine the mean difference between the SI and estimated value of SI derived by sSI from the regression equation mentioned above. It was necessary to convert sSI to the same scale as SI using the procedure noted previously since TOST compares mean difference between two groups. In a TOST, equivalency is determined when the 90% confidence interval (CI) of mean difference is settled within a predetermined equivalence margin [33]. Since the equivalence margin between 0.25 and 0.5 of effect size adjusted for standard deviation is usually chosen in practice [34], we chose 0.25 of the standardized effect size of the SI as the equivalent margin. Power values of 0.8 were considered statistically significant. Equivalence tests were also performed for 14 patient subgroups including aged, non-aged, female, male, trauma, pregnant, acute myocardial infarction, sepsis, chronic respiratory disease, intracranial disease, hypotension, normotension, hypertension, and severe hypertension. The potential risk of type I errors due to multiple subgroup analyses would be expected to occur in up to 0.7 nominally statistically significant interaction tests (*P* < 0.05) by chance alone, which was a sufficiently low possibility [35].

Finally, we performed a sensitivity analysis to validate robustness with respect to missing data with an equivalence test using all cases that were not missing any data [36]. R software, version 3.4.1 (The R Foundation, Vienna, Austria) was used for all statistical analyses.

## Results

### Characteristics of the study subjects

**Fig. 1:**
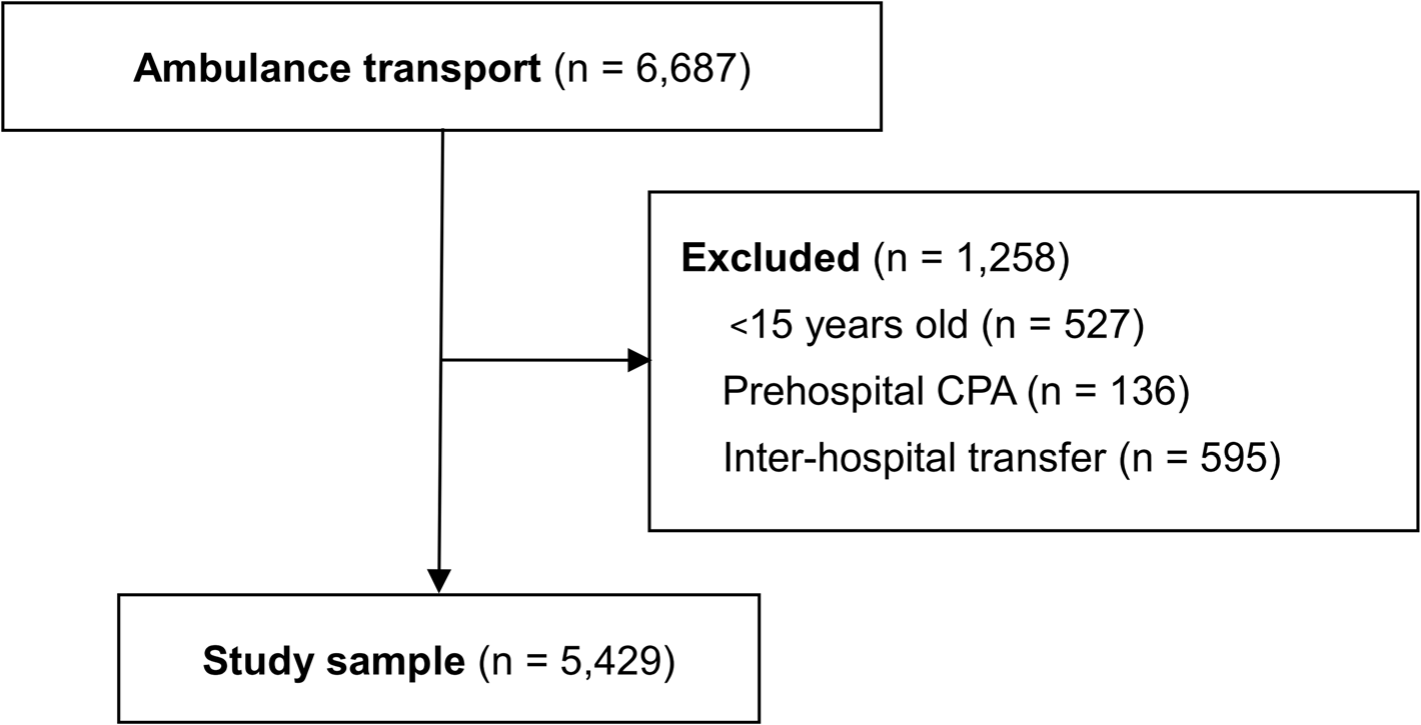
Flowchart of the study

There were 6687 patients who were transported to the two hospitals via ambulance during the study period; 1258 of these patients were excluded including 527 aged < 15 years, 136 who experienced prehospital cardiopulmonary arrest, and 595 who underwent inter-hospital transfer. Thus, 5429 patients were ultimately evaluated (Fig. 1). The patients’ characteristics are shown in Table 1. The median age was 68 (IQR 47–81), and the median ages of those aged 15–64 and aged ≥65 were 43 (IQR 29–56) and 80 (IQR 73–85), respectively. The median HR and SBP values were 84 (IQR 72–97) and 140 (IQR 121–163) mmHg, respectively. Prehospital HR of 765 cases (14.1%) and prehospital SBP of 721 cases (13.3%) were substituted for missing values of the ED. Remaining missing HR and SBP values were complemented by using the multiple imputation method for 958 (17.6%) and 912 (16.8%) patients, respectively. No other data were missing.

**Table 1:** Patients’ Characteristics

	N (%)
Age, years	
15–64	2420 (44.6)
≥ 65	3009 (55.4)
Sex	
Female	2677 (49.3)
Male	2752 (50.7)
Pre-existing condition	
Trauma	1653 (30.4)
Pregnancy	91 (1.7)
Acute myocardial infarction	97 (1.8)
Sepsis	65 (1.2)
Chronic respiratory disease	114 (2.1)
Intracranial disease	643 (11.8)
Systolic blood pressure	
Hypotension (< 90 mmHg)	129 (23.8)
Normotension (90–139 mmHg)	2467 (45.4)
Hypertension (140–179 mmHg)	2282 (42.0)
Severe hypertension (≥180 mmHg)	551 (10.1)

## Main results

**Fig. 2:**
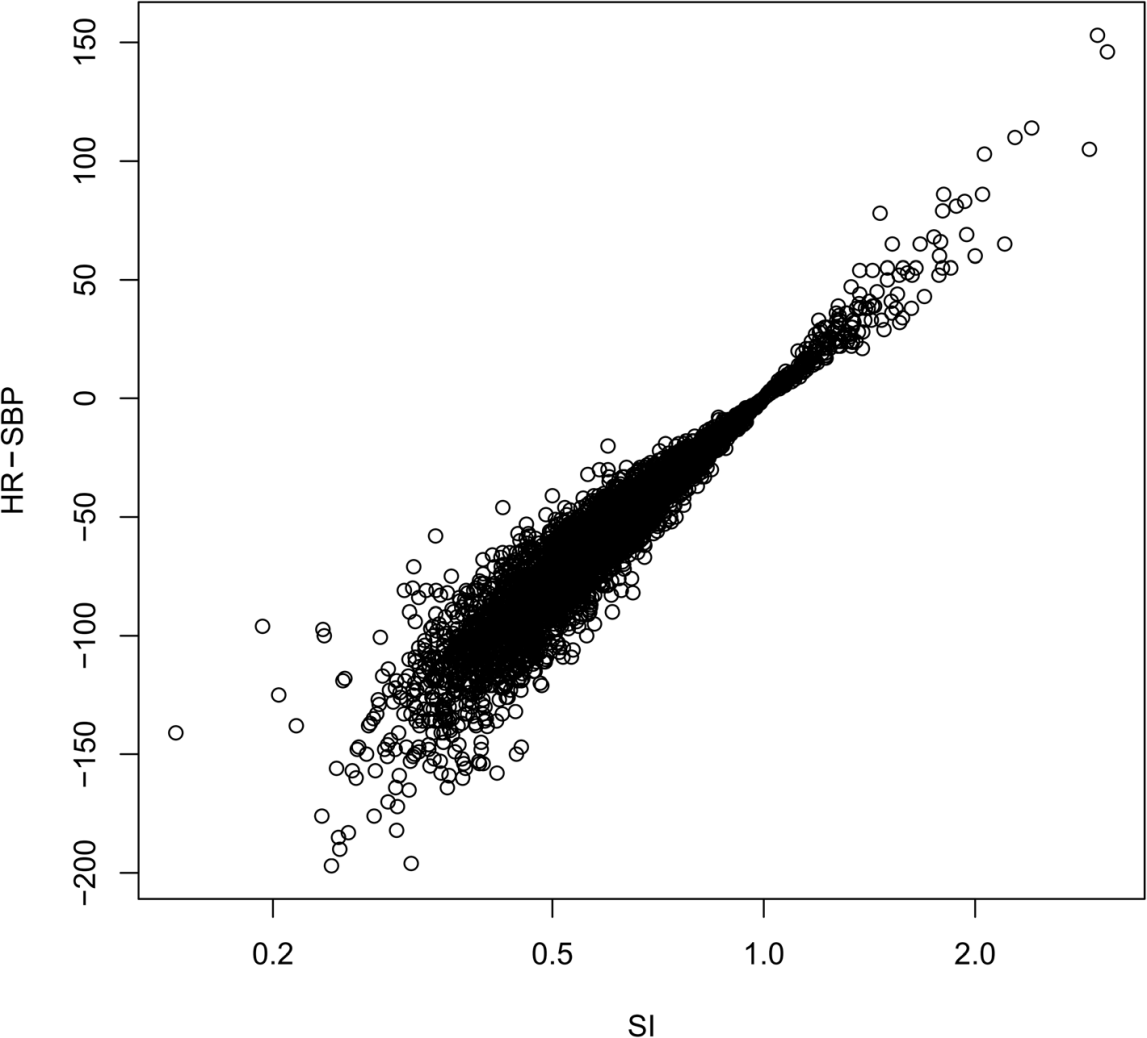
Correlation plot of the SI and sSI (HR − SBP). SI: shock index, sSI: simple shock index, HR: heart rate, SBP: systolic blood pressure. SI plotted on logarithmic scale

The Pearson’s product-moment correlation coefficient between the SI and sSI was 0.917 (95% CI 0.912–0.921, *P* < .001), indicating an extremely high correlation. The log SI/sSI correlation plot represented a proportional relationship (Fig. 2) and the regression equation was estimated as sSI = 258.55 log SI using the least-squares method with logarithmic transformation. According to this equation, an sSI value of − 12 was found to correspond to the SI value of 0.9.

**Fig. 3:**
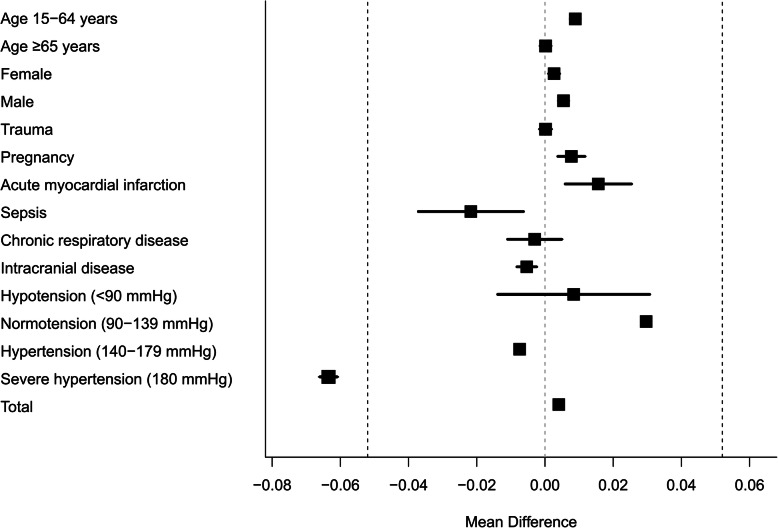
Equivalency tests between SI and SI derived by sSI (HR − SBP). SI: shock index, sSI: simple shock index, HR: heart rate, SBP: systolic blood pressure. Equivalence margin is 0.052. SI derived by sSI was calculated according to the following estimation equation: sSI = 258.55 log SI

Next, an equivalence test with TOST was performed for comparisons between SI and the estimated value of SI derived by sSI. The estimated value of SI was calculated as 10^sSI/258.55^ according to the regression equation mentioned above. Equivalence margin was determined as ±0.052, since it was 0.25 of the standardized effect size of SI. The TOST revealed an equivalency between the SI and sSI (mean difference, 0.004; 90% CI, 0.003 to 0.005; statistical power, 100.00%). Similar consequences were also derived from subgroup analyses except for severe hypertension (Table 2, Fig. 3). The statistical power for all analyses of each subgroup and all subjects were over 97.29%.

**Table 2:** SI and SI derived by sSI (HR-SBP) equivalence tests

	N	Mean difference (90% CI)
Age, years		
15–64	2420	0.009 (0.008 to 0.011)*
≥ 65	3009	0.000 (− 0.001 to 0.002)*
Sex		
Female	2677	0.003 (0.001 to 0.004)*
Male	2752	0.005 (0.004 to 0.007)*
Pre-existing condition		
Trauma	1653	0.000 (−0.002 to 0.002)*
Pregnancy	91	0.008 (0.004 to 0.012)*
Acute myocardial infarction	97	0.016 (0.006 to 0.026)*
Sepsis	65	−0.022 (− 0.038 to − 0.006)*
Chronic respiratory disease	114	−0.003 (− 0.011 to 0.005)*
Intracranial disease	643	−0.005 (− 0.008 to − 0.002)*
Systolic blood pressure		
Hypotension (< 90 mmHg)	129	0.008 (−0.014 to 0.031)*
Normotension (90–139 mmHg)	2467	0.030 (0.029 to 0.031)*
Hypertension (140–179 mmHg)	2282	−0.007 (− 0.009 to − 0.006)*
Severe hypertension (≥180 mmHg)	551	−0.064 (− 0.066 to − 0.061)
Total	5429	0.004 (0.003 to 0.005)*

*SI* Shock index, *sSI* Simple shock index, *HR* Heart rate, *SBP* Systolic blood pressure, *CI* Confidence interval. Equivalence margin is 0.052. SI derived by sSI was calculated according to the following estimation equation: sSI = 258.55 log SI.

*Significant results based on the 90% CI values

In the sensitivity analysis, the robustness of equivalency was validated using 3629 cases (66.8%) that did not lack any data of ED (mean difference was 0.006, 90% CI 0.004 to 0.007, statistical power 100.00%).

## Discussion

In this study, we revealed that the sSI, which was derived by subtracting the SBP from the HR, strongly correlated with the SI among the patients transported via ambulance. Meanwhile, an sSI value of > **−** 12 was observed to correspond to the known SI cut-off value of > 0.9, which is the most common optimized cut-off point, as has been previously described [16]. However, as positive numbers are more easily calculated and interpreted, instead of calculating the sSI cut-off using HR − SBP > − 12 as we have done in the present study, we recommend using either SBP **−** HR < 12 or HR + 12 > SBP instead, which can be considered to be equivalent to an SI > 0.9.

This finding confirms the utility of using the sSI for more rapid assessment of the condition of patients admitted for emergency care than the more complicated SI. Assuming that a calculator is not available, judging whether the SI is more than 1.0 is quite easy because the hypothesis is true when the value of HR is greater than that of SBP. However, when the SI cut-off value is 0.9, the judgement becomes difficult. As calculating HR/SBP mentally can cause confusion, we often calculate SBP times 0.9 mentally and then compare it with HR. For example, when an HR of 103 and SBP of 114 are known, we calculate 114 × 0.9 = 102.6 and then compare it with 103. Thus, we can judge SI to be > 0.9 because 103 > 102.6. Obviously, this procedure is complicated and tends to lead to miscalculation. On the other hand, an alternative criterion of sSI > − 12 makes the procedure much simpler. For example, using the same values of HR of 103 and SBP of 114, calculating HR plus 12 is the first step to solve the hypothesis. When the sum (e.g. 103 + 12 = 115) is compared with SBP, we are able to judge that sSI is > − 12 because 115 > 114. Since this addition is quite easy, mental calculation can be performed at a glance.

While attempts have been made to improve the predictive ability of SI for mortality or other outcomes, these endeavours have made the process more complicated. Examples of such previously proposed predictors include an index called ‘age shock index’ derived by multiplying the SI with the patient’s age, or another referred to as the ‘modified shock index’ obtained by dividing the HR by the mean blood pressure [19, 20]. Other complicated predictors were also proposed such as ‘respiratory adjusted shock index’ calculated by multiplying the SI with the respiratory rate/10 and ‘reverse shock index multiplied by Glasgow Coma Scale score’ derived by dividing the Glasgow Coma Scale by the SI [21, 22]. To the best of our knowledge, this is the first study aimed at simplifying the calculation using subtraction, as no previous groups have proposed the idea of subtracting the SBP from the HR for purposes of estimating the SI.

The sSI was shown to be equivalent to SI in most subgroups by TOST and only significantly underestimated SI in the severe hypertension subgroup. However, we believe this finding will have minimal on the utility of sSI in clinical practice mainly because SI was mainly developed to identify patients who were in a critical state despite their vital signs being within normal range [16, 31]. Another reason is that very low SI values should not be ignored as they can often pose risks to patients contrary to what is expected. Several studies have reported a J-shaped relationship between SI and mortality [4, 13], that is mortality increases when the SI value is under 0.5. Therefore, our new indicator sSI would not be utilized on patients with severe hypertension who would typically require close monitoring as their SI values tend to be very low.

Regarding the moderate number of missing values, when comparing analysis using values generated from the imputation method and from using only cases without missing values our results indicated good concordance between the measures examined and indicated that sSI is a useful and precise tool.

This study has several limitations. We were unable to investigate patients of different ethnicities because this study was conducted in a single geographic area. Moreover, there is a dearth of a theoretical framework to support the sSI, given that this study was intended as merely a proposal of a pragmatic alternative to the SI. Additionally, although the SI is used to predict mortality, necessity of blood transfusion, or necessity for intensive care unit admission, our study did not address these outcomes; we tested only the correlation between the SI and sSI here. Moreover, we could not prove that the assessed patients were clinically in shock or not since this diagnosis is determined using various factors such as cardiac output, lactate level, urine output, blood gas analyses, and mental status. Furthermore, there is certainly a possibility of multiplicity in the subgroup analyses, although this possibility was estimated to be sufficiently low. These issues should be addressed in future studies intended to clarify the scientific underpinnings of sSI, to further validate sSI as an accurate substitute calculation for SI, or to justify the clinical utility of sSI.

## Conclusions

The sSI was demonstrated to highly correlate with the SI among patients transported to hospitals in our study via ambulance, though further studies are needed to validate its clinical utility. Given that the sSI is easier to calculate and use for performing evaluations, it can be a useful and highly precise alternative to the SI.

## Data Availability

The datasets generated and/or analysed during the current study are available in the Open Science Framework repository, https://osf.io/n94zs/ or DOI 10.17605/OSF.IO/N94ZS.

## Abbreviations

SI: Shock index
HR: Heart rate
SBP: Systolic blood pressure
sSI: Simple shock index
ED: Emergency department
IQR: Interquartile range
TOST: Two one-sided test
CI: Confidence interval

## References

[1] Allgower M, Burri C (1967). Shock index. Dtsch Med Wochenschr.

[2] Sloan EP, Koenigsberg M, Clark JM, Weir WB, Philbin N (2014). Shock index and prediction of traumatic hemorrhagic shock 28-day mortality: data from the DCLHb resuscitation clinical trials. West J Emerg Med.

[3] Cannon CM, Braxton CC, Kling-Smith M, Mahnken JD, Carlton E, Moncure M (2009). Utility of the shock index in predicting mortality in traumatically injured patients. J Trauma.

[4] Odom SR, Howell MD, Gupta A, Silva G, Cook CH, Talmor D (2016). Extremes of shock index predicts death in trauma patients. J Emerg Trauma Shock.

[5] Schroll R, Swift D, Tatum D, Couch S, Heaney JB, Llado-Farrulla M (2018). Accuracy of shock index versus ABC score to predict need for massive transfusion in trauma patients. Injury..

[6] McNab A, Burns B, Bhullar I, Chesire D, Kerwin A (2012). A prehospital shock index for trauma correlates with measures of hospital resource use and mortality. Surgery..

[7] Bruijns SR, Guly HR, Bouamra O, Lecky F, Wallis LA (2014). The value of the difference between ED and prehospital vital signs in predicting outcome in trauma. Emerg Med J.

[8] Nathan HL, AEl A, Hezelgrave NL, Seed P, Butrick E, Miller S (2015). Shock index: an effective predictor of outcome in postpartum haemorrhage?. BJOG.

[9] Nathan HN, Seed PT, Hezelgrave NL, Greeff AD, Lawley E, Anthony J (2019). Shock index thresholds to predict adverse outcomes in maternal hemorrhage and sepsis: a prospective cohort study. Acta Obstet Gynecol Scand.

[10] Abe N, Miura T, Miyashita Y, Hashizume N, Ebisawa S, Motoki H (2017). Long-term prognostic implications of the admission shock index in patients with acute myocardial infarction who received percutaneous coronary intervention. Angiology..

[11] Wang Q, Shen H, Mao H, Yu F, Wang H, Zheng J (2019). Shock index on admission is associated with coronary slow/no reflow in patients with acute myocardial infarction undergoing emergent percutaneous coronary intervention. J Interv Cardiol.

[12] McCall SJ, Musgrave SD, Potter JF, Hale R, Clark AB, Mamas MA (2015). The shock index predicts acute mortality outcomes in stroke. Int J Cardiol.

[13] Myint PK, Sheng S, Xian Y, Matsouaka RA, Reeves MJ, Saver JL (2018). Shock index predicts patient-related clinical outcomes in stroke. J Am Heart Assoc.

[14] Berger T, Green J, Horeczko T, Hagar Y, Garg N, Suarez A (2013). Shock index and early recognition of sepsis in the emergency department: pilot study. West J Emerg Med.

[15] Middleton DJ, Bedford R, Neilly M, Myint PK, Smith TO (2019). Shock index predicts outcome in patients with suspected sepsis or community-acquired pneumonia: a systematic review. J Clin Med.

[16] Rady MY, Smithline HA, Blake H, Nowak R, Rivers E (1994). A comparison of the shock index and conventional vital signs to identify acute, critical illness in the emergency department. Ann Emerg Med.

[17] Kamikawa Y, Hayashi H (2019). Predicting in-hospital mortality among non-trauma patients based on vital sign changes between prehospital and in-hospital: an observational cohort study. PLoS One.

[18] Koch E, Lovett S, Nghiem T, Riggs RA, Rech MA (2019). Shock index in the emergency department: utility and limitations. Open Access Emerg Med.

[19] Zarzaur BL, Croce MA, Magnotti LJ, Fabian TC (2010). Identifying life-threatening shock in the older injured patient: an analysis of the National Trauma Data Bank. J Trauma.

[20] Liu Y, Liu J, Fang ZA, Shan G, Xu J, Qi Z (2012). Modified shock index and mortality rate of emergency patients. World J Emerg Med.

[21] Caputo N, Reilly J, Kanter M, West J (2018). A retrospective analysis of the respiratory adjusted shock index to determine the presence of occult shock in trauma patients. J Trauma Acute Care Surg.

[22] Kimura A, Tanaka N (2018). Reverse shock index multiplied by Glasgow coma scale score (rSIG) is a simple measure with high discriminant ability for mortality risk in trauma patients: an analysis of the Japan trauma data Bank. Crit Care.

[23] Graham LN, Smith PA, Stoker JB, Mackintosh AF, Mary DA (2004). Sympathetic neural hyperactivity and its normalization following unstable angina and acute myocardial infarction. Clin Sci.

[24] Barcroft H, Edholm OG, McMichael J, Sharpey-Schafer EP (1944). Posthaemorrhagic fainting: study by cardiac output and forearm flow. Lancet..

[25] Lamantia MA, Stewart PW, Platts-Mills TF, Biese KJ, Forbach C, Zamora E (2013). Predictive value of initial triage vital signs for critically ill older adults. West J Emerg Med.

[26] Chester JG, Rudolph JL (2011). Vital signs in older patients: age-related changes. J Am Med Dir Assoc.

[27] Eccles SR, Subbe C, Hancock D, Thomson N (2014). CREWS: improving specificity whilst maintaining sensitivity of the National Early Warning Score in patients with chronic hypoxaemia. Resuscitation..

[28] Haukoos JS, Newgard CD (2007). Advanced statistics: missing data in clinical research —part 1: an introduction and conceptual framework. Acad Emerg Med.

[29] Newgard CD, Haukoos JS (2007). Advanced statistics: missing data in clinical research —part 2: multiple imputation. Acad Emerg Med.

[30] World Health Organization (2002). Proposed working definition of an older person in Africa for the MDS Project.

[31] Vandromme MJ, Griffin RL, Kerby JD, McGwin G, Rue LW, Weinberg JA (2011). Identifying risk for massive transfusion in the relatively normotensive patient: utility of the prehospital shock index. J Trauma.

[32] Williams B, Mancia G, Spiering W, Rosei EA, Azizi M, Burnier M (2018). 2018 ESC/ESH guidelines for the management of arterial hypertension: the task force for the management of arterial hypertension of the European Society of Cardiology (ESC) and the European Society of Hypertension (ESH). Eur Heart J.

[33] Walker E, Nowacki AS (2010). Understanding equivalence and noninferiority testing. J Gen Intern Med.

[34] Chow S, Shao J, Wang H, Lokhnygina Y (2017). Sample size calculations in clinical research.

[35] Wang R, Lagakos SW, Ware JH, Hunter DJ, Drazen JM (2007). Statistics in medicine — reporting of subgroup analyses in clinical trials. N Engl J Med.

[36] Thabane L, Mbuagbaw L, Zhang S, Samaan Z, Marcucci M, Ye C (2013). A tutorial on sensitivity analyses in clinical trials: the what, why, when and how. BMC Med Res Methodol.

